# The effect of health status and living arrangements on long term care models among older Chinese: A cross-sectional study

**DOI:** 10.1371/journal.pone.0182219

**Published:** 2017-09-07

**Authors:** Liangwen Zhang, Yanbing Zeng, Ya Fang

**Affiliations:** 1 State Key Laboratory of Molecular Vaccinology and Molecular Diagnostics, School of Public Health, Xiamen University, Xiamen, Fujian, China; 2 Key Laboratory of Health Technology Assessment of Fujian Province University, School of Public Health, Xiamen University, Xiamen, Fujian, China; University of West London, UNITED KINGDOM

## Abstract

**Background:**

Currently, there are many studies focusing on the influencing factors of the elderly people’s living arrangements or health status, but little is known about the relationship between living arrangements or health status and long-term care models for the old-age, especially the joint effects.

**Objective:**

We aimed to assess the effects of health status and living arrangements on long-term care models (LTCM) among the elderly of Xiamen, China, especially their cumulative joint effects.

**Methods:**

A total of 14,373 participants aged ≥ 60 years by multistage sampling in Xiamen of China were enrolled. Multinomial logistic regression was used to estimate the Odds ratios (ORs) regressing LTCM on health status and living arrangements using the Anderson model as theoretical framework.

**Results:**

Totally, 14,292 valid questionnaires were obtained, of which 86.37% selected home care. With the increase of disability degree, older people are more likely to choose institutional care, compared to living alone (ORs = 1.75, 2.06, 4.00, 4.01 for the “relatively independent’, “mild disability’, “moderate disability’, and “total disability’, respectively, in comparison with “completely independent’). The elderly living with children and other family members preferred to choose home care. (ORs = 0.50, 0.39, 0.40, and 0.43 for the “living with children’, “living with spouse’, “living with children and spouse’, and “living with others’, respectively, in comparison with “alone’). Additionally, residence, number of children, education level, and feelings of loneliness were the determinants of the choice of social pension.

**Conclusion:**

A multitude of older people are trended to choose home care in Xiamen of China. There was an interaction and joint effect between the degree of disability and the living arrangements on LTCM. Therefore, policymakers should pay close attention to care for those living alone, childless, and disabled elders to meet their care needs, especially in home care. In addition, the social construction of facilities for elders in rural areas should be strengthened.

## Introduction

The phenomenon of population aging is a serious social problem around the world, especially in China, the most populous nation. Population aging and long-term care (LTC) for elderly are global concerns that no longer occur in developed countries only. China became an “aging society” in 2000, and has experienced an unprecedented process of aging in its population [1–3]. In 2014, there were 212 million people who were 60 years of age and 138 million who were 65 years of age in China, accounting for 15.5% and 10.1% of the total population, respectively [4]. It was estimated that the proportion of individuals aged 60 years and 65 years in China will exceed 30% and 20% respectively by 2050 [5–7]. The demand for LTC is increasing, because of the aging of the population and the increase in chronic and degenerative diseases [8]. A hefty challenge of caring for the elderly population is faced by all the countries, especially in China. Therefore, investigating the willingness of the elderly pension has paramount significance for improving the long-term care system.

Long term care models (LTCM) for older persons have become increasingly crucial policy concerns in developing countries and rapidly aging Asia, especially in China. Generally, there are three models of LTCM: the traditional family care model was serviced by family members (home care), community provides care services living in the home or community (community care), and living in nursing home (institutions pension) [9]. Traditionally, elder care has been provided by adult children at home under the cultural norm of *xiao* (or filial piety), the central value of Chinese family relationships. The implementation of one-child policy and rapid socioeconomic transformation, however, have led to much doubt on whether family, especially adult children, will be able to care for such a large number of elderly population [8]. In the family structure, the traditional family in the gradual disintegration, and was replaced by “4-2-1” structured family (a family with 4 grandparents, 2 parents and a single child in a family), “elderly couple family” and “empty nest” family. As the ability of the family to provide care for their elderly members has been undermined by the shrinking of family size and tendency toward nuclear family type, families no longer possess the capacity to provide adequate care. According to previous studies, although adult children are not necessarily less indoctrinated to the cultural tradition of *xiao*, it is financially and physically too difficult and constrained for them to care for their elderly parents with dual demands of work and caring for their own offspring[10]. Moreover, large numbers of elderly parents are left behind at home in impoverished villages due to the massive scale of rural–urban migration. Consequently, compared to urban counterparts, Chinese elders lived in rural areas that are located in a much more vulnerable position.

Although the function of the family care was gradually weakening and the family care model was not adapted to the situation of aging. Data shows that the proportion of elderly people choose home care was 95% in the United Kingdom, 96.3% in the United States, 96.2% in Sweden, 98.6% in Japan, 94% in Singapore, 72.2% in Thailand, and 75.4% in China [10]. With the weakening of the function of the family care, especially for the elderly living alone cannot take care of themselves, the need for institutional care and community care may become increasingly strong. The quality of institutional care is superior but the cost is high, and there is a contradiction between supply and demand [11]. The community care can save cost and service flexibility, but there are an army of problems such as the small service type, ambiguous subject, and the unformed scale effect [12]. In the face of the status quo that three kinds of LTCM have their own advantages and disadvantages, how to understand the choice of LTCM for the elderly, and then according to the service object to optimize the three kinds of LTCM, providing scientific theoretical basis for decision makers, is a significant and meaningful issue.

Previous studies have shown that disability in Activities of Daily Living (ADL) positively influences living arrangements and further affects the elderly LTCM selection [13–15]. Disability degree in ADL is an adverse outcome of frailty that brings a burden on frail elderly people, care providers and the care system. Disability elderly people need an excellent support system from the family or society to protect. For example, the higher degree of disability of people, the higher degree of their long-term care, and they are more likely to live together with their spouses or children or seek assistance from community nurses or live in nursing home [16]. Moreover, previous research suggested that the living arrangements of older persons played a key role in their use of formal and informal care and in their health and well-being. Elderly living alone may need more care from the community or institutional support and help due to loneliness or low life satisfaction. Therefore, we propose two research hypotheses: 1) the health status of the elderly will have an impact on their LTCM. The higher degree of disability the elderly has, the higher probability they incline to select the community or institutional care. 2) The living arrangements of the elderly will have an impact on their LTCM option, and the disability degree and living arrangements may exist joint effects.

The Anderson model has been reported by several studies on elderly utilization of health services [17,18], health-related quality of life [19], and long-term care services [20]. It shows the use of health services by predisposing (e.g. age, education, gender, and race), enabling (e.g. family income, marital status, kinship network, social support network) and need components (e.g. health status, ADL). However, there is a little literature using Anderson model as a theoretical framework for LTCM. In this study, the model is used to analyze the associations of predisposing, enabling and need variables with use of different LTCM among the elderly.

Despite the well-studied individual associations of living arrangements with human health status and the impact factors with living arrangements, limited studies have uncovered the associations of living arrangements and health status with elderly LTCM option, especially the complexity of their interrelationship. Consequently, this study aim to examine Xiamen elderly disability circumstances, living conditions and pension will, study the influencing factors of the elderly LTCM selection using the Anderson model as the theoretical framework, and focuses on the influence of self-care ability, the living conditions of the elderly LTCM selection, which provide a theoretical basis for the policy making.

## Materials and methods

### Study population and sampling

A cross-sectional survey entitled “The Investigation on the Status Quo of Health and Long-Term Care for the Elderly in Xiamen City” was performed across 173 communities in Xiamen, China, in 2013. A total of 261,043 individuals were aged ≥ 60 years in Xiamen at the survey time and about 5.5% of overall elderly populations were covered in this survey. The participants were enrolled by a multistage sampling procedure. In the first stage, all 38 subdistricts in Xiamen were selected. In stage 2, one-third of communities were randomly sampled from each subdistrict and a total of 173 communities were included in the end. The randomization of these communities was performed by computer-generated random numbers. In stage 3, participants were conveniently selected from each community by controlling for gender and age composition.

The number of individuals to be sampled in each community was determined according to its proportion of eligible older adults. Finally, 14,292 effective participants aged ≥ 60 years were enrolled, who were local household registered by multistage stratified sampling procedure, with a response rate of 99.44%.

### Measures

The dependent variables were the three primary LTCMs in China. In this study, the LTCM was self-reported by the participants based on the question “Where do you most want to receive long-term care?” The answer options offered were family care, community care, and institutional care.

Living arrangements were assessed by one question: “What is your current living arrangement?” The answers were included five categorized: alone, living with children, living with spouse, living with children and spouse, and living with others (nephew, niece, son-in-law, nanny etc.).

Health status was measured by a modified Katz Activities of Daily Living Scale, which included six basic activities of daily living (ADL): eating, toileting, bathing, dressing, getting in and out of bed and mobility. Each item had three response choices: “completely independent”, “needing some help”, and “completely dependent”. If any answer was “completely dependent”, participants were defined as disabled; otherwise the participants were categorized as nondisabled. By this scale, health status was classified into five categories: completely independent, relatively independent, mild disability, moderate disability, total disability [21].

**Fig 1:**
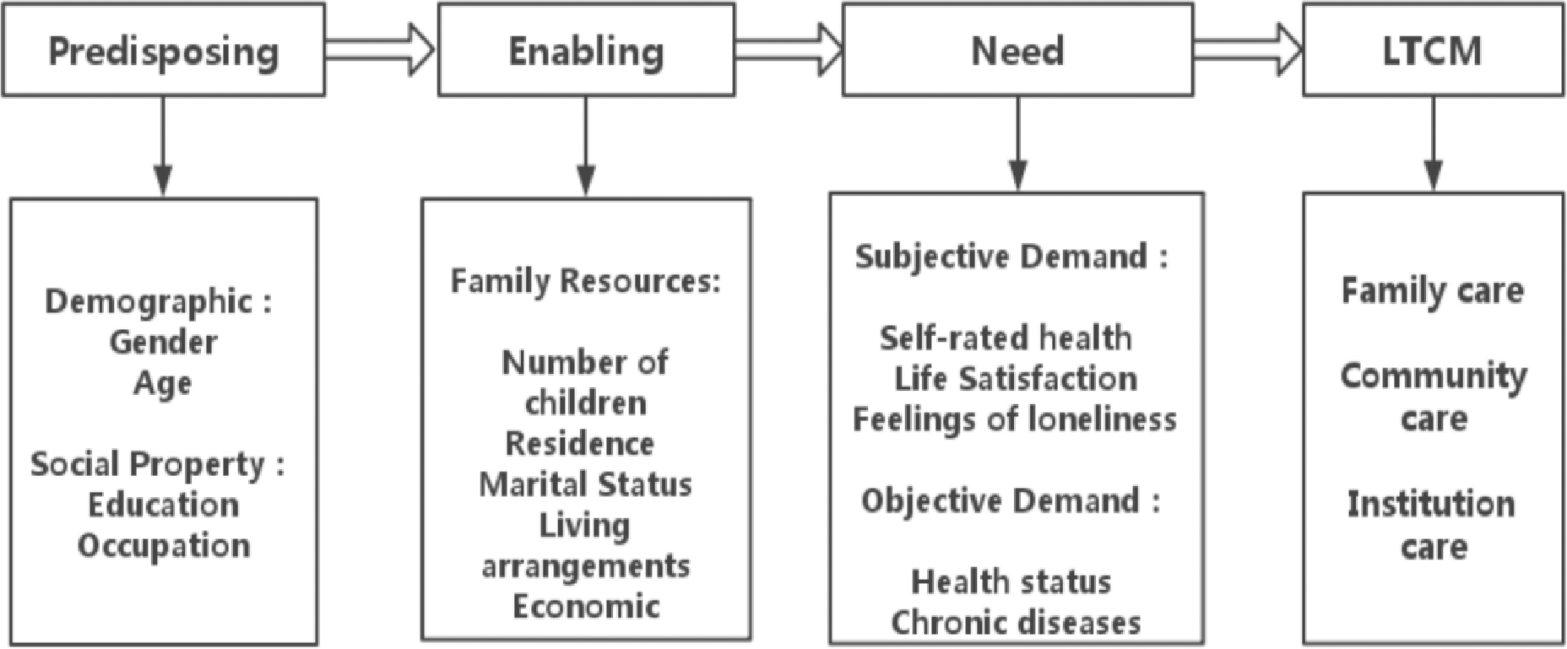
The Anderson theoretical model of influencing factors on LTCM.

On the basic of the Anderson behavior model, this paper constructed the adjusted Anderson theoretical model of influencing factors on LTCM according to the research purpose and variable information availability (Fig 1). The predisposing variables were age, gender, occupation and education. The enabling variables were expressed by the living arrangements, residence location, marital status, medical insurance, family income and number of Children. The need variables were evaluated by ADL, chronic diseases, the self-rated health, life satisfaction and the feelings of loneness.

### Ethics statement

The study protocol was approved by the ethical review committee of School of Public Health, Xiamen University. All participants read a statement that explained the purpose of the survey and provided written informed consent before participation in the study.

### Statistical analysis

Descriptive statistics were used for health status, living arrangements, predisposing, enabling characterization and need indicators by calculating the proportion of distribution in each stratum of LTCM. We illustrated the distribution of age by the three LTCM and the distribution of three LTCM by the disability degree and living arrangements, respectively, using violin plot with jittering points. The interspersion of participants jointly by disability degree and living arrangements was shown by a bubble plot, in which the association between health status and living arrangements can be intuitively presented. Chi-square (*Χ*^*2*^) tests were used to identify the group differences in proportions and one-way ANOVA F-tests for continuous variables. One-way ANOVA F-tests were applied to identify significant differences between different health status and LTCM. In order to reduce sample selection bias and control for competing risks, we included all sample members in the analysis. All key variables were coded as multi-category variables. Multinomial logistic regressions were used to examine whether health status and living arrangements predict the LTCM, respectively, controlling covariates in ModelⅠ(Tables 1–3). Then we tested the main effects and interaction effect between health status and living arrangements on LTCM in Model II, respectively (Tables 1–4). Finally, we further tested the total effect of health status and living arrangements on LTCM, controlling for other covariates. The ORs of total effect is the ORs product of the main effect and the interaction effect in Model Ⅲ. Data entry was performed independently and discrepancies were checked by two trained research assistants using Epidata software version 3.1 (Epidata Association, Odense, Denmark). All statistical analyses were conducted using SPSS 16.0, SAS 9.3 and R3.2.1. A value of *P*< 0.05 was considered statistically significant.

**Table 1:** Basic characteristic of 14,292 participants according to the LTCM.

	Family n(%)	Community n(%)	Institution n(%)	Total n(%)	*Χ*^*2*^	*P-value*
**Health status (n = 14292)**						
Completely independent	10930(88.5)	1285(83.5)	284(69.4)	12499(87.5)	182.30	0.000
Relatively independent;	950(7.7)	160(10.4)	73(17.8)	1183(8.3)		
Mild disability	124(1.0)	19(1.2)	8(2.0)	151(1.1)		
Moderate disability	79(0.6)	25(1.6)	9(2.2)	113(0.8)		
Total disability	261(2.1)	50(3.2)	35(8.6)	346(2.4)		
**Living arrangements (n = 14285)**						
Alone	1420(10.0)	221(14.4)	121(29.7)	1582(11.1)	205.25	0.000
Spouse	3585(29.1)	512(33.3)	104(25.5)	4201(29.4)		
Children	2481(20.1)	236(15.3)	64(15.7)	2781(19.5)		
Spouse&Children	4303(34.9)	478(31.1)	93(22.8)	4874(34.1)		
Others	730(5.9)	91(5.9)	26(6.4)	847(5.9)		
**Gender (n = 14292)**						
Male	5883(47.7)	813(52.8)	192(46.9)	6888(48.2)		
Female	6461(52.3)	726(47.2)	217(53.1)	7404(51.8)	14.90	0.001
**Age (n = 14292)**						
60~69	6036(48.9)	682(44.3)	174(42.5)	6892(48.2)	17.51	0.002
70~79	3971(32.2)	530(34.4)	145(35.5)	4646(32.5)		
80 or above	23377(18.9)	327(21.2)	90(22.0)	2754(19.3)		
**Occupation (n = 14240)**						
None	1572(12.8)	166(10.8)	61(15.1)	1799(12.6)	15.57	0.004
Farmer	5192(42.2)	684(44.6)	141(34.9)	6017(42.3)		
Non-farmer	5539(45.0)	683(44.6)	202(50.0)	6424(45.1)		
**Education (n = 14204)**						
Illiterate	4042(32.9)	487(32.0)	130(31.9)	4659(32.8)	22.16	0.005
Primary	3802(31.0)	491(32.3)	108(26.5)	4401(31.0)		
Junior high school	2358(19.2)	258(17.0)	73(17.9)	2689(18.9)		
Senior high school	1334(10.9)	179(11.8)	66(16.2)	1579(11.1)		
College and above	738(6.0)	107(7.0)	31(7.6)	876(6.2)		
**Residence (n = 14292)**						
Urban	6108(49.5)	673(43.7)	211(51.6)	6992(48.9)	19.32	0.000
Rural	6236(50.5)	866(56.3)	198(48.4)	7300(51.1)		
**Marital status (n = 14292)**						
Married	8626(69.9)	1069(69.5)	216(52.8)	9911(69.3)	54.27	0.000
Unmarried	3718(30.1)	470(30.5)	193(47.2)	4381(30.7)		
**Number of children (n = 14282)**						
0	204(1.7)	68(4.4)	74(18.1)	346(2.4)	507.66	0.000
1	1863(15.1)	192(12.5)	78(19.1)	2133(14.9)		
2	3247(26.3)	430(28.0)	96(23.5)	3773(26.4)		
≥3	7022(56.9)	848(55.1)	160(39.2)	8030(56.2)		
**Medical insurance (n = 14291)**						
Yes	12243(99.2)	1517(98.6)	408(99.8)	14168(99.1)	0.58	0.446
No	100(0.8)	22(1.4)	1(0.2)	123(0.9)		
**Economic status (n = 14284)**						
Income exceeded expenditures	3270(26.5)	501(32.6)	153(37.4)	3924(27.5)	51.33	0.000
Balance	6510(52.8)	746(48.5)	166(40.6)	7422(52.0)		
Expenditures exceeded income	2557(20.7)	291(18.9)	90(22.0)	2938(20.6)		
**Having chronic diseases (n = 14292)**						
Yes	7722(62.6)	1030(66.9)	312(76.3)	9064(63.4)	41.30	0.000
No	4622(37.4)	509(33.1)	97(23.7)	5228(36.6)		
**Self-rated health (n = 14292)**						
Bad	2109(17.1)	326(21.2)	135(33.0)	2570(18.0)	99.01	0.000
Fair	5957(48.3)	752(48.9)	193(47.2)	6902(48.3)		
Good	4278(34.7)	461(30.0)	81(19.8)	4820(33.7)		
**Life Satisfaction (n = 14287)**						
Bad	649(5.3)	111(7.2)	67(16.4)	827(5.8)	124.69	0.000
Fair	4847(39.3)	682(44.3)	172(42.1)	5701(39.9)		
Good	6843(55.5)	746(48.5)	170(41.6)	7759(54.3)		
**Feelings of loneliness (n = 14226)**						
Often	390(3.2)	102(6.7)	67(16.5)	559(3.9)	255.49	0.000
Sometimes	4161(33.9)	541(35.3)	174(42.9)	4876(34.3)		
Seldom/Never	7736(63.0)	890(58.1)	165(40.6)	8791(61.8)		

**Table 2:** Multinomial logistic models of LTCM (ModelⅠ).

	Community			Institution		
	Model 1	Model 2	Model 3	Model 1	Model 2	Model 3
**Health status**						
Completely independent	1.00		1.00	1.00		1.00
Relatively independent	1.11(0.91,1.35)		1.13(0.93,1.38)	1.70(1.23,2.36)**		1.75(1.26,2.43)***
Mild disability	1.15(0.71,1.92)		1.25(0.75,2.07)	1.70(0.79,3.66)		2.06(0.95,4.46)
Moderate disability	2.14(1.32,3.46)**		2.38(1.47,3.85)***	3.11(1.47,6.55)**		4.00(1.88,8.50)***
Total disability	1.30(0.93,1.82)		1.39(0.99,1.95)*	3.31(2.11,5.19)***		4.01(2.53,6.35)***
**Living Arrangements**						
Alone		1.00	1.00		1.00	1.00
Spouse		0.98(0.73,1.32)	0.96(0.71,1.29)		0.50(0.29,0.84)**	0.45(0.26,0.76)**
Children		0.58(0.47,0.71)***	0.56(0.46,0.69)***		0.39(0.28,0.55)***	0.35(0.25,0.49)***
Spouse&Children		0.81(0.60,1.09)	0.78(0.58,1.05)		0.40(0.23,0.68)***	0.35(0.20,0.59)***
Others		0.80(0.60,1.08)**	0.77(0.57,1.04)		0.43(0.26,0.72)**	0.35(0.21,0.59)***
**Predisposing**						
Gender(female)	1.09(0.97,1.24)	1.08(0.96,1.22)	1.09(0.96,1.22)	0.87(0.69,1.09)	0.81(0.65,1.02)	0.83(0.67,1.04)
Age(year)	1.01(1.00,1.02)***	1.01(1.00,1.02)***	1.01(1.00,1.02)**	1.01(0.99,1.02)	1.01(0.99,1.03)	1.00(0.99,1.01)
Occupation(farmer)	1.16(1.04,1.30)**	1.18(1.05,1.32)**	1.18(1.05,1.32)**	1.04(0.86,1.26)	1.07(0.88,1.30)	1.05(0.87,1.28)
Education(Illiterate)	1.12(1.05,1.19)***	1.12(1.05,1.19)***	1.12(1.05,1.19)***	1.27(1.14,1.42)***	1.28(1.15,1.43)***	1.28(1.14,1.43)***
**Enabling**						
Residence (rural)	0.61(0.54,0.70)***	0.62(0.53,0.72)***	0.62(0.53,0.72)***	0.69(0.53,0.91)**	0.72(0.55,0.95)*	0.74(0.56,0.97)*
Marital status (Unmarried)	1.09(0.94,1.25)	0.91(0.70,1.18)	0.92(0.71,1.19)	0.64(0.50,0.83)	0.88(0.54,1.43)	0.89(0.55,1.46)
Number of children(0)	0.88(0.82,0.95)***	0.90(0.84,0.97)**	0.92(0.84,0.97)**	0.54(0.48,0.60)***	0.57(0.51,0.64)***	0.57(0.51,0.64)***
Economic status (income< expenditures)	0.90(0.83,0.98)*	0.88(0.81,0.96)**	0.89(0.81,0.97)**	1.07(0.91,1.27)	0.99(0.84,1.17)	1.06(0.90,1.25)
**Need**						
Chronic diseases (no)	1.88(1.78,1.99)*	1.88(1.78,1.98)*	1.88(1.78,2.00)*	2.01(1.65,2.91)**	2.10(1.85,2.90)**	2.01(1.85,2.71)**
Self-rated health (bad)	0.99(0.90,1.08)	0.95(0.87,1.04)	0.99(0.90,1.09)	0.86(0.72,1.03)	0.72(0.61,0.85)***	0.86(0.71,1.03)
Life Satisfaction (bad)	0.88(0.80,0.98)*	0.88(0.79,0.98)*	0.89(0.81,0.98)*	0.88(0.73,1.06)	0.85(0.71,1.03)	0.89(0.74,1.08)
Feelings of loneliness (Often)	0.87(0.78,0.97)**	0.88(0.79,0.97)*	0.89(0.80,0.99)*	0.61(0.51,0.73)***	0.62(0.52,0.75)***	0.66(0.55,0.80)***

Note

* P<0.05

**P<0.01.

***:P<0.001

Reference variable = Home

**Table 3:** Odds ratios (95% confidence intervals) obtained from multivariable adjusted^a^ multinomial logistic models (Model Ⅱ).

	Model 1	Model 2	Model 3	Model 4
**Health status**				
Completely independent	1.00		1.00	1.00
Relatively independent	1.24(1.04,1.47)**		1.26(1.06,1.51)**	1.64(1.19,2.26)**
Disability	1.68(1.34,2.10)***		1.85(1.48,2.32)***	3.95(2.31,6.74)***
**Living Arrangements**				
Alone	1.00	1.00		1.00
Spouse	0.83(0.64,1.08)	0.80(0.61,1.04)		0.83(0.63,1.09)
Child	0.51(0.43,0.61)***	0.49(0.41,0.58)***		0.54(0.44,0.66)***
Spouse&Children	0.67(0.52,0.88)**	0.64(0.49,0.83)***		0.67(0.51,0.89)**
Others	0.68(0.52,0.88)**	0.63(0.49,0.82)***		0.60(0.44,0.83)***
Health status* Living Arrangements				
Completely independent* Alone				1.00
Relatively independent* Spouse				0.79(0.52,1.21)
Relatively independent* Child				0.60(0.38,0.94)*
Relatively independent* Spouse&Child				0.60(0.37,0.98)*
Relatively independent* Others				0.46(0.21,0.98)*
Disability* Spouse				0.85(0.42,1.73)
Disability* Child				0.79(0.41,1.54)
Disability* Spouse&Child				0.97(0.49,1.95)*
Disability* Others				2.25(1.07,4.73)*
Model *Χ*^*2*^	301.11***	345.87***	374***	391.10***
Nagelkerke R^2^	0.60	0.61	0.62	0.62

Note

*P<0.05

**P<0.01.

***P<0.001

Y1 = family care

Y2 = Socialization of LTCM(community care or institutional care)

^a^All models used the same set of covariates: predisposing variables (gender, age, occupation, education), enabling variables (residence, marital status, number of children, economic status), need variables (chronic diseases, self-rated health, life satisfaction, feelings of loneliness).

**Table 4:** Odds ratios (95% confidence intervals) obtained from multivariable adjusted^a^ multinomial logistic models regressing LTCM on health status and living arrangements, accounting for interactions of self-care level and living arrangements. (Model Ⅲ).

Health Status	Living Arrangement				
	Alone	Spouse	Child	Spouse & child	Others
Completely independent	1.00	0.69(0.58,0.81)	0.47(0.39,0.57)	0.54(0.46,0.64)	0.53(0.40,0.70)
Relatively independent;	2.68(1.99,3.58)	1.23(0.90,1.69)	0.65(0.46,0.90)	0.75(0.51,1.10)	0.55(0.23,1.08)
Disability	2.94(1.76,4.93)	1.33(0.84,2.11)	0.86(0.59,1.24)	1.19(0.77,1.85)	3.35(2.15,5.23)

## Results

### Descriptive analyses

The descriptive characteristics of the study sample are shown in Table 1. In the 14,292 participants (6,888 males, 48.19%), the mean age was 71.49±8.34 years. The proportions of the elderly self-care situation were 87.45%, 8.28%, and 4.27%, for the completely independent, relatively independent and disability respectively. The proportions of mild disability, moderate disability, and total disability were 56.72%, 18.53% and 24.75%, respectively, accounting for 1.06%, 0.79% and 2.42% of the total elderly population. The main caregivers of the elderly were spouses and sons, accounting for 39.07% and 30.77%, respectively. The participants (32.98%) whose number of family members was 5 or more have accounted for, and the elderly living with their spouses and children together accounted for 34.12%, followed by living with spouse (29.41%) and living alone (11.07%). For the selection of LTCM, 86.37%% of the elderly wished live at home, and only 2.86% chose to live in the pension institutions, 10.77% for community care. Besides, with the increase of the degree of disability, the proportion of elderly people in the selection of community and institution increased to 22.1% and 8.57%, respectively.

**Fig 2:**
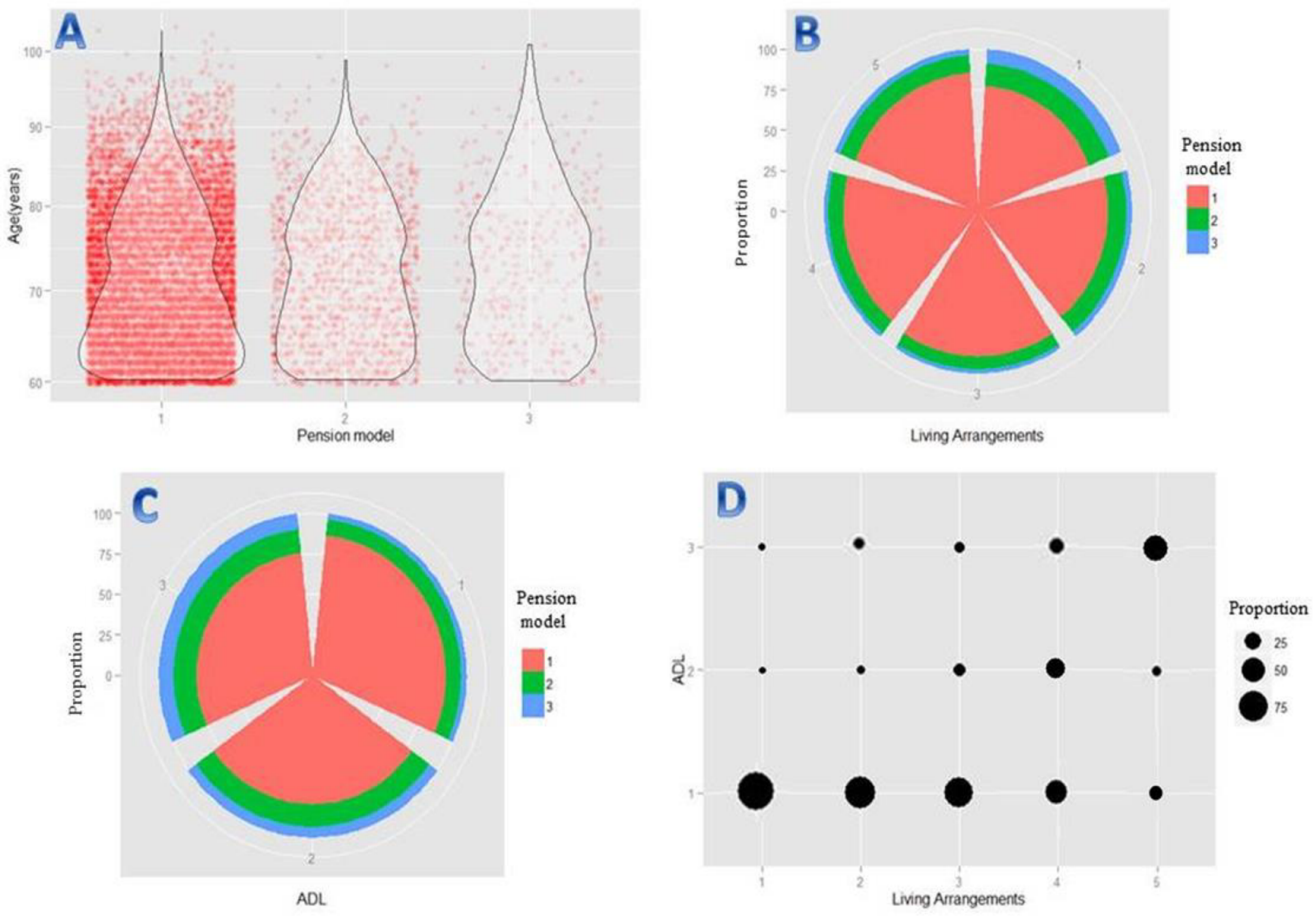
**Violin plots with jittering points (A) and Sector chart with proportion (B, C) of participants’ LTCM under age, living arrangements and ADL, and bubble plot (D) for interspersion of participants by living arrangements and ADL.** For LTCM, 1 = home care, 2 = community care, 3 = institution care. For living arrangements,1 = alone,2 = with spouse,3 = with children,4 = with spouse and children,5 = with others. For health status, 1 = totally independent, 2 = relatively independent, 3 = disability.

Participant distributions for age, five living arrangements, three health statuses and three LTCMs were shown in Fig 2. (1) Fig 2**A** presented the age distribution among three LTCMs by violin plot with jittering points. The number of jittering points was the number of participants and the violin curves were rotated kernel density curve to display the probability density. In this survey, 87.45% of the elderly choose home care, and the age is mainly distributed in the 60–80 years. With the increase of age, the choice of the community or institutional care showed a rising trend. (2) The distribution of LTCM is different in different ways of living was shown in Fig 2B. The proportion of elderly individuals living alone to choose community care and institutional care regardless of home care is relatively high, accounting for 14% and 7.6%, respectively. Compared with community care and institutional care, the proportion of selecting home care in different ways of living arrangements is higher, and about 89.2% of the elderly living with spouse and children choose home care to be taken care. (3) Fig 2**C** demonstrated the distribution of LTCM under different health conditions. From the picture, we can figure out that the proportion of elderly of totally independent choosing home care is up to 87.4%, compared to other LTCM, but with the increase of disability degree, the proportion selecting the community and institutional care is also rising. (4) Finally, the bubble plot of living arrangements and health status was shown in Fig 2**D**. There is a correlation between the degree of disability and the living style. The health status of elderly individuals living alone is relatively well than others, and with the change of old people's living style, the ability to take care of themselves has gradually declined. The elderly folks who live with their children and spouse have a relatively high degree of disability, which is most obviously in the situation of the elderly living with others (mainly including the health care workers, nannies, etc.).

### Multinomial logistic regressions

The multinomial logistic models for LTCM are presented in Table 2. It is consistent with our hypothesis, health status and living arrangements are significantly associated with LTCM in all models adjustment for other covariates. There was an increasing trend of odds as health status attainment improved no matter whether living arrangements entered the model or not (Models 1 and 3). Specifically, those moderate disability, total disability, living with children, and living with others are more likely to choose community-based home service at Model 1–3, compared with those living in the home. With the increase of disability degree, older adults are more likely to choose institutional care, compared to living alone. Living with elderly relatives or children and other family members prefer home care. Furthermore, residence, the number of children, education level, whether suffering from chronic diseases, loneliness affects both the pension community and institutional care choices. Among them, those who were older, living in the rural, and non-farming, and had fewer the number of children, higher education level, more kinds of chronic diseases, poorer life satisfaction, and stronger feelings of loneliness are more likely to select the community care. The elderly with living in the countryside, being unmarried, having fewer number of children, having the higher the education level, having chronic diseases, and having stronger feeling of loneliness were more likely to choose institutional care.

Table 3 showed the odds ratios (95% confidence intervals) from multinomial logistic models. The results showed that the living arrangements and health status were statistically significant, and there was interaction effect in the case of the model 1–4 with adjustment for covariates. Inside, the elderly of disability and not living with their families were obviously tended to choose socialization of LTCM (community care or institutional care), and with the increase of the disability degree, the trend was more obvious. On the contrary, the old people with good health condition and living together with the spouse or children were more inclined to the home care.

The multinomial logistic models for LTCM are shown in Table 4. The elderly who had “disability’ and were living with others presented the highest odds of wishing getting long-term-care from community and institution, followed by the elderly with “disability’ and living alone. In addition to living with their children, the disabled elderly individuals tend to choose the community or institution pension. Elderly folks living alone or living with their spouses, in the case of relatively independent and disability may choose community or institutional care. What’s more, the old people with completely independent are willing to take home care no matter whom they living with.

## Discussion

The findings in this study showed that health status and living arrangements were associated with LTCM, however, a few previous studies had found that the factors that were significantly related to the selection of institutional elder care in a separate study, which was no longer significant in this study, such as age, gender [22–24]. The health status of elderly individuals (including disability, chronic disease, self-rated health, life satisfaction, and loneliness), living arrangements and their combined effect were significant on the elderly LTCM option. Moreover, the marital status, residence, education, and number of children also had a significant impact on the LTCM of the elderly. Because of the survey population, sample size, the region was different, so there may be appear such result. In addition, the age and gender may not have much impact on the choice of institutional care for the elderly. The elderly might be more important to their own health status, the existing care supply situation, and the quality of service. Older people are more concerned about their own health status, the existing care supply, and the quality of service, currently.

For the selection of LTCM, 86.37%% of the elderly were willing to live at home, and only 2.86% chose to live in the pension institutions, 10.77% for community-based home care. Consistent with previous findings, “Home care” is still the preferred LTCM for most of older individuals [25], because they may be familiar with the community environment, neighborhood, social networks they have formed in the long term. However, with the development of social and economic and the change of family structure, the care pattern has some new characteristics of the times, and the community care and institutional care developed rapidly. Along with the increase of the disability degree, the proportion of home care for the elderly is gradually declining such as 87.4%, 82.1%, and 75.4% for the home care at completely independent, mild disability, and total disability, respectively, and the proportion of selection of the community or institution pension is gradually increasing. For example, 22.1% old people choose the community care under the condition of moderate disability. Compared to previous research results (9.8%), the proportion had a significant increase [26].

Community care has a geographical sense of belonging, sense of participation, and psychological identity, which can solve the problems encountered in the old life in time, reduce their psychological pressure and labor burden of their children. Moreover, it provides professional care services to make up for the lack of family care resources, so that the elderly enjoy the social support services at home. In addition, although a certain degree of elderly living in the place of life has a variety of options, such as the home for the Elderly in the rural areas, apartment for the aged, nursing home, social welfare institutes. However, due to the traditional concept, economic conditions, and the characteristics of the elderly psychological needs, institutional care is still occupy the smallest proportion of LTCM, which is consistent with the results of the previous studies [27,28]. It is worth mentioning that the quality of service in China's institutional care is very worrying, currently. The quality of life in some institutions can be truly bleak. Many pension agencies lack of professional medical care, rehabilitation and health care services due to lack of health human resources.

Disability degree, chronic diseases, self-rated health and loneliness were significantly influenced on the choice of long-term care for the elderly. The disabled elderly in dressing, eating, bathing and other aspects are in need of help, and community care provides a series of services such as housework, home health care, home delivery, emergency call, and security assistance for elderly who cannot take care of themselves. The old adults can live in their familiar environment, and obtain the social care service. Therefore, from modelsⅠcan be seen that consistent with the hypothesis, older individuals with high levels of disability can have a higher degree of acceptance of community care in comparison to home care.

The elderly people with chronic diseases, the worse the self-rated health, the more intense the loneliness are more inclined to choose institutional care. In other conditions unchanged, the probability of selecting the institution pension for elderly who have chronic diseases is two times higher than ones without chronic diseases. Because of their poor health condition and ADL, in addition to the need to receive daily life care, they also need to receive professional guidance in the prevention of chronic diseases, health counseling, and rehabilitation care. Such as the need for long-term retention of gastric tube and tracheal cannula, biliary drainage tube or suffering from a variety of serious chronic disease and paralysis, hemiplegia and life cannot take care of disability in the elderly. Home care is far from being able to meet the needs of this population.

Living alone has an overwhelmingly significant effect on the choice of institutional care for elderly people. It is consistent with the previous hypothesis that people who live alone cannot be independent of their health status and their daily life. Because of the availability and accessibility of family resources, they are more likely to choose the institutional care. In other conditions unchanged, the proportion in the choice of institutional person for elderly folks living alone is about 3 times more than family care. In addition, there is a significant common effect on LTCM for elderly people between the living arrangements and the disability degree. Compared with living alone, the elderly characters living with their spouses and children and other relatives are more willing to choose the home care. With the rise of the degree of disability, they began to choose the pension agency getting more professional care services, which is especially obvious for old-age folks living with their spouses. The disabled elderly people with their children living together are still prone to select home care, which has a great relationship with Chinese traditional ideas raise children to provide against old age. Furthermore, similar to previous studies [29], this study also found that older people living in rural areas more likely to choose community or institutional care. The reason probably may be due to the faster urbanization in recent years, which led to a large number of migrant workers into the city. Because of the lack of necessary life care and comfort, elderly people living alone are willing to accept community or institutional care.

The number of children has a significant impact on the choice of home care for the elderly. Consistent with the hypothesis, the less number of children the elderly had, the less care resources they received, and they are more likely to choose institutional care. In addition, this is deeply influenced by the concept of “filial piety” in traditional Chinese culture. Many elderly people with children will be misconstrued as being unfilial to their children. The level of education also has a significant effect on the choice of long-term care for the elderly. Generally speaking, compared to low educational level of the elderly, high cultural degree elderly people are more open-minded, the concept “raise children to provide against old age”is relatively weak, and social participation consciousness is strong, which is conducive to the understanding and acceptance the new affair of social care. In addition, the elderly with high level of education tend to have a high social status, favorable economic situation, a strong ability to pay, and a higher degree of recognition of social care.

At present, the trinity of the LTCM including the home care, community home care, and institutional care will be an inevitable trend in the future of the aging society. Currently, 5.09% of individuals are willing to enter the nursing home, the old apartments and other social pension institutions. And the survey results show that people's pension will be influenced by a host of factors and constraints, but can be attributed to three aspects: self-factors (health, concept), economic factors and environmental factors (internal and external home environment). If any aspect of these three aspects is changed, it can bring diverse chooses for LTCM. Along with the concept of people endowment change, empty nest family increases, the social pension system construction and perfect, which makes growing people to choose in different endowment patterns, based on their own pension needs.

There are several limitations associated with this study. First, the data in our study were a cross-sectional survey and this limited the interpretation of our results, making it hard to draw causal conclusions. Second, the participants were sampled from one city in China, which may be local characterized and in turn exist some bias for interpretation in countrywide. Third, this study uses information on self-health related reporting. Health status of the elderly only uses the basic activities of daily living (BADL) scale to evaluate. Since the health of the elderly is comprehensive and multi-dimensional, it should be combined with subjective and objective indicators to conduct a comprehensive evaluation of the health status of the elderly. Therefore, longitudinal studies are needed to conduct to assess policy trends and key stakeholders. In particular, we hope to further explore the relationship between different fertility norms and LTCM selection of the elderly in the future.

## Conclusions

In conclusion, LTCM shows the demands of the different long-term care needs of the elderly in certain degree. A multitude of older people are inclined to choose family care in Xiamen of China. Both the health status and living arrangements were associated with LTCM for elderly people. Higher disability degree, living in rural, living alone, and childless was observed to be associated with selecting institutional care and community care. Therefore, policymakers should keep close attention to care for those living alone, childless, and disabled older adult populations to meet their care needs, preferentially. On one hand, the government should formulate the criterion and system of home care services; improve care personnel training of care workers, and increase capital investment to meet the LTC needs of the majority of elderly. Moreover, the government should provide equitable, accessible, high quality health care services involving health management, disease care and rehabilitation care in different LTCM. On the other hand, the government should enhance the attractiveness of social pension for the elderly by enriching community and institutional care service content, improving service quality, and strengthening publicity and education, to break the social prejudices of institutional care. In addition, the policymakers should focus on strengthening the social construction of facilities for the elderly in rural areas.

## Supporting information

S1 File Supporting data. (XLS)
